# An analysis of physical vulnerability to flash floods in the small mountainous watershed of Aceh Besar Regency, Aceh province, Indonesia

**DOI:** 10.4102/jamba.v10i1.550

**Published:** 2018-04-26

**Authors:** Azmeri Azmeri, Amir H. Isa

**Affiliations:** 1 Department of Civil Engineering, Syiah Kuala University, Indonesia; 2 Regional Office of Public Works, Division of Irrigation, Indonesia

## Abstract

Vulnerability is a loss rate perceived from elements at risk owing to the impact of hazards on determined magnitude and frequency. Vulnerability assessment of elements at risk is a critical component in the risk assessment framework. Considerable studies regarding flash floods have been conducted, but the depth of information on vulnerability is still limited. This study presents a model of the physical vulnerability of buildings caused by the incidence of flash floods, which is strengthened through intensity of the impact process and loss. This model used a non-linear regression approach using behavioural data in the form of the propagation height of the flood. The order-2 polynomial distribution can represent the relationship between the magnitudes of the best process and loss rate. The results are the risk assessments of an exposed area in mitigation strategies.

## Introduction

A flash flood disaster has the characteristics of a fast water flow, with a flood hydrograph peak time of fewer than 6 hours. The phenomenon of flash floods often occurs in areas with a steep slope and small rainfall catchment. This condition leads to a precipitation response of a short runoff and can cause landslides (Tao & Barros 2013). Another factor causing flash floods is the extreme rainstorm upstream the watershed. Climate change boosting intense rainstorms can increase the frequency of the occurrence of the flash floods and landslides, raising the possibility of a secondary disaster of the natural dam (Chen, Lin & Chen 2015). The flood is affected by geomorphometric of the land slopes, the erosion and material sediment (Borga et al. 2014). The deposit material from river bank collapse can trigger the formation of natural dams. Aside from being able to play a role in increasing the capacity to reduce the risk of the flood disaster, the dam can also become a new hazard factor.

Rainfall with a heavy flow discharge and earthquakes are the main causes of the collapse of the natural dam. Dam failure can lead to a new disaster in the form of flash floods. The water stored in the pool rea of the dam will flow with great speed and discharge downstream. The inability of the capacity of the dam to accommodate the river flow will cause overflow to the right and left of the river bank. This overflow can inundate areas downstream of dams that are densely populated. Analysis can show the risk of disaster in the affected areas (Wirustyastuko & Nugroho 2013).

Flash floods led to land degradation and resulted in the destruction of property. It is a widely distributed worldwide phenomenon leading to more economic disasters (Nkeki, Hannah & Ojeh 2013). The flash flood calamity has a significant social and economic impact with loss of life and homes, incurring high costs for recovering damages (Rosso & Rulli 2002).

Flash flood is an interaction between people and environment (Hualou 2011). The flooding research has focused the consequences: damage to human lives and property, health hazards, clean-up costs, traffic obstruction, economic loss and infrastructure damage (Okereke 2007).

Threats by flash flood risks are the calculation of the hazards event intensity, frequency and the vulnerability of the exposed humans and elements. The vulnerability assessment requires the identification of risk factors due to flash floods. It is useful for the management strategies toward the flash floods risks (Karagiorgos et al. 2016a).

Many methods to calculate vulnerability, which can be quantitative, semi-quantitative or qualitative (Fuchs, Kuhlicke & Meyer 2011). These models collect data after an event, by Totschnig, Sedlacek and Fuchs (2011), for flash floods, and for river floods (Kreibich et al. 2010). Many researches use the assessment of vulnerability for housing (Papathoma-Kohle et al. 2012; Totschnig et al. 2011) or Kreibich et al. (2010); Seifert et al. (2010) for commercial buildings. The assessment of vulnerability is a calculation of environmental exposure. According to Karagiorgos et al. (2016a), the physical vulnerability is a relation between intensity of the process and water depth of flooding was implied the DoL (Degree of Loss).

The assessment of vulnerability refers to the environment, economic or social vulnerabilities. It is based on engineering sciences and the economics are related to the calculation of monetary damages on assets. (Fuchs et al. 2011).

The flash floods have repeatedly occurred in Aceh province, Indonesia. Over the last 30 years, there have been four flood disaster events (1987, 2000, 2013 and 2016) at the Krueng Teungku watershed of the Aceh Besar Regency. Based on earlier research (Azmeri, Yulianur & Listia 2015), the flood occurred in the Seulimeum district on 07 January 2016, with an accumulated rainfall of 294 mm per day.

The disaster had a significant impact on Beurenuet village and the surrounding areas, and the deluge was the water damming at the Krueng Teungku watershed. The main cause is collapse of a natural dam at the upstream (Azmeri, Hadihardaja & Vadya 2016). Azmeri et al. (2015) stated that the collapse of the said natural dam was caused by overtopping, that is, the heavy flow of water through the top of the dam causing erosion and avalanches.

As a result of the repeated threat of flash floods and damages resulting from the disaster, the aim of the study was to formulate a model of the physical vulnerability of buildings caused by the incidence of flash floods. It is focused through vulnerability functions that describe the relationship between the level of risk for the loss and intensity of the process. The results are the risk assessments of an exposed area in mitigation strategies.

## Location of study

**FIGURE 1:**
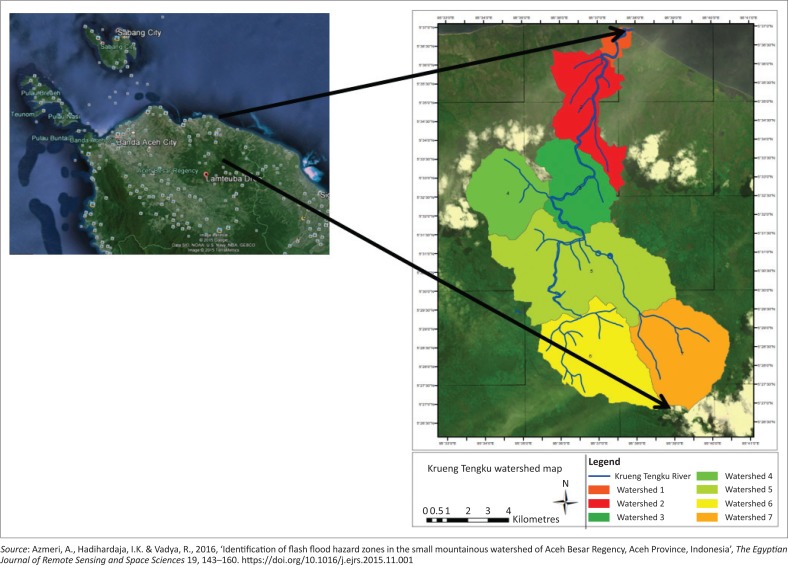
The Location of study – Krueng Teungku drainage watershed.

The location of study is located at the Krueng Teungku watershed of Aceh Besar Regency, Aceh province, at coordinates 5°26’40’’ – 5°38’20’’ North Latitude and 95°32’30’’ – 95°40’50’’ East Longitude (Figure 1).

From wet climatic conditions, topography, river and slope morphology, and soil types, this field is prone to floods and landslides. This situation is more vulnerable when there is a transitional function of green land upstream, which provides the potential for collapse of damming at the Krueng Teungku watershed, if there is high rainfall intensity. Based on the field survey, from the surface geological condition of Krueng Teungku watershed, the potential for a landslide is very much evident. The situation is further exacerbated by several points on the river channel that are naturally damaged.

## Method

According to Fuchs et al. (2007), the vulnerability is the relationship between the damage ratio and intensity of the process. Based on Keiler, Zischg and Fuchs (2006), exposed buildings are evaluated by assigning a monetary value to the building. In this study, the loss data were also measured by using an economic approach of the reconstruction value of each element that was exposed. Therefore, information about elements at risk of exposure in the study area is required, as well as the intensity data of flash flood events. These values were obtained for each building associated with the intensity of the respective process. These data were analysed using a regression approach, to infer the function of vulnerability that serves as the building’s structural constraint about floods in the Krueng Teungku watershed. The vulnerability curve links the loss data with high propagation caused by the flash floods.

The characteristics of the exposed building were recorded using empirical data collection between April and June 2017 through field survey. These components obtained are the type of building, area and the building materials used. These values are based on the size of the building by using the average value for each of its parts. The calculation is based on the Regulation of the Minister of Public Works and People Housing Number: 28/PRT/M/2016 concerning the unit price analysis of work field of public works. For the price of material, wages and equipment, it refers to the rate decided by the Governor of Aceh regarding the Material Unit Price for Public Works and Services for the needs of the Provincial Government of Aceh Darussalam of 2016, and in Aceh Besar Regency according to the study site. The monetary values that include reconstruction materials and equipment, as well as wages for labour in the study area, are given in Table 1.

**TABLE 1:** The monetary values of reconstruction materials and equipment for housing.

Number	The reconstruction materials and equipment	Unit	The Moneter (IDR)
1	Installation of a red brick wall (5 cm × 11 cm × 22 cm) thickness of one mixture stone 1 Portland cement: 4 Sand	1 m^2^	283 686
2	Installation of mixture stucco 1 PC: 4 S thickness of 15 mm	1 m^2^	66 904
3	Installation of floor tile (30 cm × 30 cm)	1 m^2^	244 208
4	Fabrication and installation of door frames and window frames, wood class II or III	1 m^3^	9 002 048
5	Fabrication and installation of door panels, wood type I or II	1 m^2^	737 968
6	New wall painting (1 layer of plamuur, one base coat and two layers of paint cover)	1 m^2^	26 692
7	New wood painting (1 layer of plamuur, one base coat and two layers of paint cover)	1 m^2^	65 381

*Source*: The Regulation of the Minister of Public Works and People Housing Number: 28/PRT/M/2016, 2016, *The unit price analysis of work field of public works*, Jakarta, Indonesia, viewed 15 September 2017, from http://birohukum.pu.go.id/uploads/DPU/2016/PermenPUPR28-2016.pdf and the Provincial Government of Aceh Darussalam (2016)

IDR, Indonesian rupiah.

Damage caused by floods are estimated based on flood hazard levels and risk elements by using depth damage curves (Sarminingsih et al. 2015). Damage ratio to calculate the vulnerability uses the economic value between the direct survey’s record of losses and each risk object (Hausmann 1992). Furthermore, the value obtained for each building is linked to the magnitude of the intensity of the process obtained from the model of flood propagation behaviour (Azmeri et al. 2015). The data of each object level is connected by scatter-plot. At the next stage, non-linear regression approach was used to obtain the vulnerability function and it represents the relationship between intensity of the process and the DoL (Equation 1) (Karagiorgos et al. 2016b):

DoL=f(I) [Eqn 1]

The targeted function type must meet three requirements: firstly, the value of its vulnerability should lie between zero and one (*f*: *I →* [0, 1]), secondly, the vulnerability functions should pass the original value [*f*(*I* = 0) = 0] and thirdly, the vulnerability functions increase [*I*_1_ ≤ *I*_2_ → *f*(*I*_1_) ≤ *f*(*I*_2_)]. Two functions were used in this study that reflected the vulnerability behaviour (Table 2). Based on Totschnig et al. (2011), while the magnitude of the process was relatively small, DoL increased gradually (Totschnig et al. 2011). In the scale of the medium process, the DoL increased almost linearly. And in the scale of the high process, DoL showed an asymptote with number one. Because of this behaviour, the linear function is not suitable. The parameter of water depth (W_d_) of the model presented was observed by using root-mean-squared error (RMSE) given in Equation 2:

$$\text{RMSE}=\sqrt{\frac{\sum_{i=1}^{n}{\left({\hat{y}}_{i}-y_{i}\right)}^{2}}{n}}$$ [Eqn 2]

**TABLE 2:** Vulnerability analysis functions.

Model (M)	Function	Formula	Parameters (θ)
M_1_	Order-2 polynomial	*a* * *wd*^2^ + *b* * *wd*	*a, b*
M_2_	Order-3 polynomial	*a* * *wd*^3^ + *b* * *wd*^2^ + *c* * *wd*	*a, b, c*

Where $\hat{y}$ and ${\hat{y}}_{i}$ are the modelled and observed at the time *i*, and *n* is the total of residential buildings exposed (Montesarchio, Lombardo & Napolitano 2009).

The best model selection (M*) is done by comparing the average RMSE for all models, to M* with the smallest error.

## Results and discussions

Krueng Teungku watershed topography is mountainous ranging from a medium to a steep slope. The topography of Krueng Teungku changes abruptly from the steep into interlace, including the landscape that is marked as a river torrent. The formation of these rapids can cause sudden surge jumps and provide the potential for natural damming and the occurrence of a flash flood. Agricultural land and community plantations extend and spread amidst the mountains. Land use is dominated by mixed forest, rice fields and farms.

**FIGURE 2:**
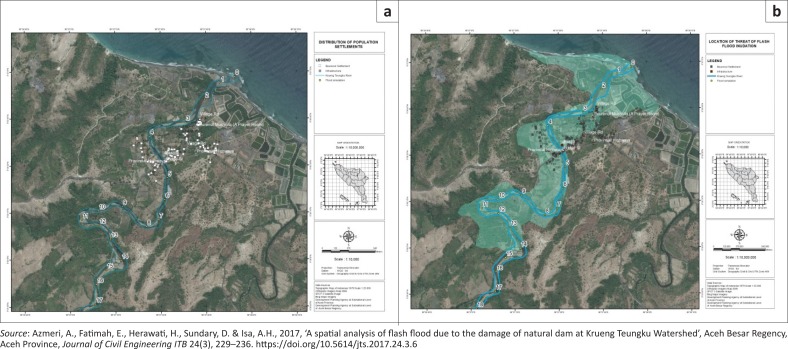
Illustration of the population and flash flood propagation. (a) Distribution of population settlements and (b) location of flash flood propagation.

In the downstream area of natural dams are the residential areas and public economic enterprises such as farming, agriculture and farms that need to be protected from the impact of flood disasters (Figure 2). So, there is an immediate need to minimise the impact of disasters through the calculation of vulnerability, especially for housing residents. It is crucial since the flash flood disaster in the Seulimeum district is a recurring disaster event.

**FIGURE 3:**
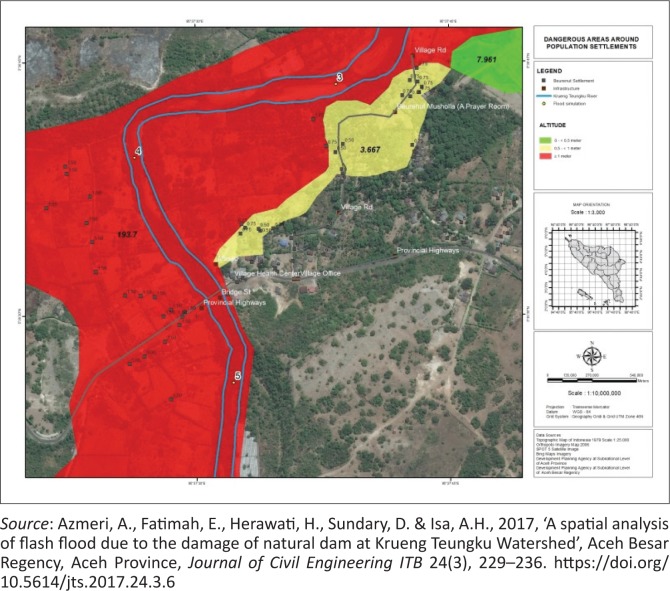
Dangerous areas around population settlements.

Based on previous research (Azmeri et al. 2017), threat areas of flood disasters at Beureunut Village are classified into three categories: Danger 1area (0.0 m – < 0.5 m), Danger 2area (0.5 m – < 1.0 m) and Danger 3area (≥ 1 m), as given in Figure 3. Based on these categories, there are 21 houses in Danger 1 area, 15 houses in Danger 2 area and two houses in Danger 3 area. As many as 38 out of 74 houses (all privately owned) were affected by a flash flood disaster.

In Table 3, for 38 houses at risk, the magnitude of the ordinary process which causes a loss is 1.09 m, and ranges from 0.25 m to 1.75 m, with a median of 1.13 m. The average damage is 18 591 612.00 IDR per unit, ranging from 185 000.00 up to 48 553 750.00 IDR, with a median of 16 156 875.00 IDR. Comparing the median which is relatively low with a high one, it shows that such there are positively tilted with only a few incidents causing high losses, while the average of total loss is quite low. The mean DoL is 0.197, ranging from 0.0019 to 0.486, with a median of 0.272.

**TABLE 3:** The statistical result of the data.

Description	Symbol	Intensity of the process (m)	Loss (IDR)	DoL (-)
Observations Number	-	38.00	-	38.0000
Minimal	*X*_(1:38)_	0.25	185 000.00	0.0019
Mean	-	1.09	18 591 612.00	0.1970
Median	-	1.13	16 156 875.00	0.2720
Maximum	*X*_(38:38)_	1.75	48 553 750.00	0.4860

DoL, Degree of loss; IDR, Indonesian rupiah.

**FIGURE 4:**
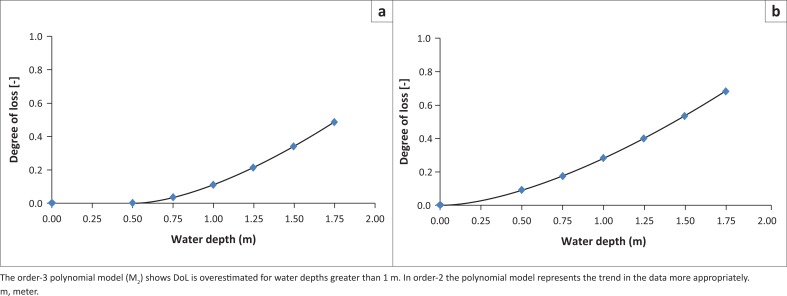
Results for the two different models. (a) The order-2 polynomial model (M_1_) and (b) the order-3 polynomial model (M_2_).

**FIGURE 5:**
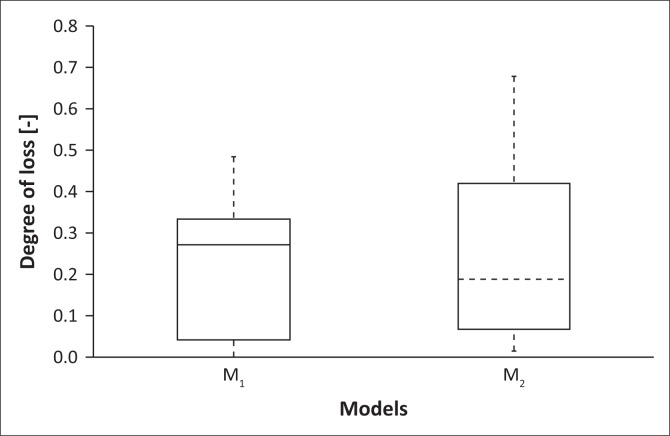
The variation of the loss rate for M_1_ and M_2_ models.

The order-2 Polynomial model (M_2_) shows a supposition that overestimation in DoL for water depth > 1 m. In contrast, the order-3 polynomial model (M_1_) does not demonstrate a systematic bias. As shown in Figure 4, the model M_1_ illustrated similar behaviour with respect to the distribution pattern of the data on models M_1_ and M_2_. The characteristic of overestimation of model M_2_ produces a higher RMSE distribution and a higher variance. The differences between M_1_ and M_2_ models are shown in the boxplots in Figure 5.

Based on the statistical results, the value of the vulnerability of buildings and calculation functions of vulnerability relies on the depth of flooding. The most effective function to describe the combined data is the order-2 polynomial distribution, which is presented in Figure 4. The average RMSE produced is shown in Table 4, and it indicates that the order-2 polynomial function has the lowest RMSE (0.0249) compared to the order-3 polynomial function (0.0688). Therefore, the order-2 polynomial function is regarded as M*.

**TABLE 4:** The root-mean-squared error result for the each model.

Model (M)	Function	Mean RMSE
M_1_	Order-2 polynomial	0.0249
M_2_	Order-3 polynomial	0.0688

RMSE, root-mean-squared error.

**FIGURE 6:**
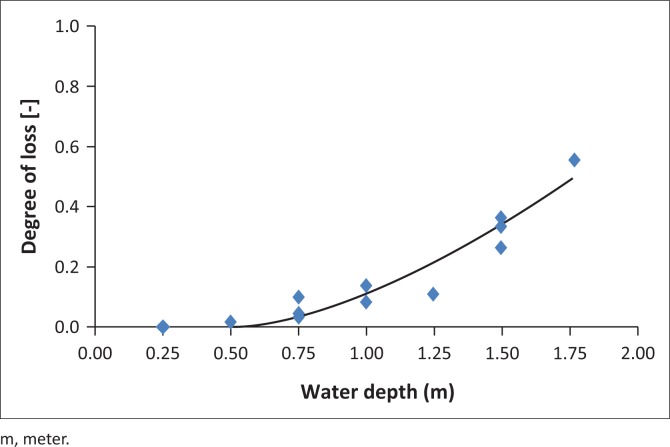
The best model of Krueng Teungku watershed for the data set.

The best model results are shown in Figure 6:

$$\text{DoL}=0.219*wd^{2}-0.1058*wd$$ [Eqn 3]

The final model is obtained by the definition of the value of vulnerability to water height, where vulnerability increases significantly. For the intensity of the process from 0.00 m to 0.25 m, the result of DoL is zero. This implies that the vulnerability is zero. At intensity of the process > 0.5 m, vulnerability and DoL increase sharply. Highest value is at DoL 0.49 for water depth 1.75 m.

## Conclusion

Because of the high number of houses lost owing to the flood disasters in the study area, it is necessary to focus on vulnerability assessment against this danger. Consequently, the vulnerability models are built with a focus on residential buildings. This method is based on data from previous studies regarding the level of danger of flash floods. Furthermore, this study links massive loss of data and the process scale.

Finally, the function of order-2 polynomial illustrates the most accurate relationship between the magnitude of the process and the loss level. The RMSE values obtained show the accuracy of the model. According to the requirements distribution, the vulnerability increased significantly by enhancing the intensity of the process. Because the buildings that were analysed were damaged by a water depth of ≤ 1.75 m, it cannot adjust the model to a higher intensity of the process. It is a limitation compared to other similar studies (Papathoma-Kohle et al. 2015; Totschnig & Fuchs 2013). However, because the flood has significantly different process characteristics in line with the sediment load, the data range must be smaller (Fuchs et al. 2011). The curve shape produced by the results was presented by Karagiorgos et al. (2016b) for residential buildings affected by the current floods.

The future needs of this study should include spatiotemporal dynamics in vulnerability to natural hazards. Over the last decade, the upstream area of Krueng Teungku watershed has transformed regarding land use change. In the downstream region, change has occurred in terms of population size, state of the economy and social characteristics that cause variations in the pattern of development. As a result, regarding spatiotemporal dynamics, the vulnerability is likely to change. The method used in this study can be applied to any region of the world where physical data is available. The results are the accurate risk assessments of an exposed area in mitigation strategies.
